# HSV-1-Specific IgG_3_ Titers Correlate with Brain Cortical Thinning in Individuals with Mild Cognitive Impairment and Alzheimer’s Disease

**DOI:** 10.3390/vaccines8020255

**Published:** 2020-05-29

**Authors:** Roberta Mancuso, Monia Cabinio, Simone Agostini, Francesca Baglio, Mario Clerici

**Affiliations:** 1 IRCCS Fondazione Don Carlo Gnocchi, 20148 Milano, Italy; mcabinio@dongnocchi.it (M.C.); sagostini@dongnocchi.it (S.A.); fbaglio@dongnocchi.it (F.B.); mario.clerici@unimi.it (M.C.); 2 Department of Pathophysiology and Transplantation, University of Milano, 20148 Milano, Italy

**Keywords:** Alzheimer’s disease, HSV-1, HSV-1-IgG subclasses, IgG_3_, mild cognitive impairment, magnetic resonance imaging (MRI)

## Abstract

Repeated reactivations of latent herpes simplex virus type-1 (HSV-1) in the central nervous system (CNS) may contribute to neurodegeneration in Alzheimer’s disease (AD) patients. Immune response is a key element for the control of viral reactivation. HSV-1 uses a number of strategies to evade immune recognition, Immunoglobulin G 3 (IgG_3_) alone counteracts humoral immunoevasion, as it is the only IgG subclass that is not blocked by the HSV-1 Fc receptor, a protein that protects virion and infected cells from antibody-mediated effector mechanisms. We examined HSV-1-specific IgG_3_ titers in serum of AD (*n* = 70) and mild cognitive impairment (MCI) (*n* = 61) subjects comparing the results to those of 67 age- and sex-matched healthy controls (HC); associations between MRI-determined brain cortical health and HSV-1-specific IgG_3_ were analyzed in a subgroup of AD and MCI subjects. HSV-1-specific IgG_3_ were more frequently detected in MCI compared to AD and HC subjects. Significant inverse correlations were found between IgG_3_ titers and brain cortical thickness in areas typically involved in dementia and HSV-1 encephalitis in AD patients; interestingly, this negative correlation was much less important in MCI subjects. All together these results suggest that in AD an inefficient IgG_3_ humoral immune response, failing to block viral replication, contributes to progressive neurodegeneration.

## 1. Introduction

Alzheimer’s Disease (AD) is the most prevalent cause of dementia, affecting 50 million of people worldwide [1] and is characterized by specific molecular hallmarks including cellular deposition of amyloid-beta and hyperphosphorylated tau in specific neuronal regions [2]. The prodromal stage of AD is referred as mild cognitive impairment (MCI); in this condition the cognitive decline is more serious that that observed in normal aging, but the symptoms are not severe enough to interfere significantly with daily life [3].

The etiology of AD is still unknown and several risk factors are suspected to favor the onset and development of the disease [4]. In recent years, the role of viral infections in the development of the AD has gained ground, and different pathogens have been suggested to be involved in the pathogenesis of the disease [5]. Amongst the viruses suspected to be associated with AD, a number of experimental and epidemiological data [6] supports the hypothesis that human herpes simplex virus type-1 (HSV-1) plays an important pathogenetic role. After primary infection, this widely-prevalent virus persists in latent state in trigeminal ganglia, with sporadic and recurrent reactivation throughout life. Upon reactivation, the virus can reach the brain, as showed by detection of its genome in brain of elderly people [7], and can cause neuronal damage, directly and/or by inducing inflammation. Notably, the ability of HSV-1 to cause an accumulation of amyloid-beta and hyperphosphorylated tau in neuronal cultures [8,9] and in an AD mouse model [10], provides a mechanistic link supporting epidemiological data. This suggests that therapeutic and/or vaccinal approaches aimed at preventing HSV-1 infection or reducing its reactivation might be useful in preventing the development of AD or could support cognitive rehabilitation in AD subjects.

Host immunity is critical to control viral reactivation, but understanding of host factors influencing the balance between viral latency and reactivation is limited [11]. It is plausible that the physiological decline of immune responses during aging [12] could impact on the frequency and the effect of viral reactivation. The immune response against HSV-1 is a complex process that involves a balance between innate and adaptive immune responses [13]. Recent results showed that significantly higher HSV-1-specific IgG titers are present in AD patients compared to age-matched healthy controls [14], and that the volume of temporal and orbitofrontal cortices positively correlate with HSV-1-specific IgG titers [15]. Taken together these results suggest a possible protective role of HSV-1-specific humoral immunity in AD. Subsequent results indicated that IgG subclasses are differently distributed in AD and in MCI patients [16]. Thus HSV-1-specific IgG_3_ were significantly more abundant in MCI compared to AD patients. However, in sera of AD patients alone, in vitro neutralization of HSV-1 was defective despite the presence of high IgG_3_ titers. These findings were somehow surprising, as IgG_3_ is endowed with the strongest neutralization capacity and is the only subclass that is not blocked by the HSV-1 Fc receptor (vFcR) [17], a protein that binds the Fc domain of IgGs and protects virions and infected cells from antibody-mediated effector mechanisms [18]. Here we seek to complement our previous works by verifying whether anti-HSV-1 IgG_3_ titers are related to neurodegeneration. To this end we correlated serum HSV-1-specific IgG_3_ with structural magnetic resonance imaging (MRI) analyses considering target brain areas typically affected in AD and in HSV-1-associated acute encephalitis.

## 2. Materials and Methods

### 2.1. Patients and Controls

One-hundred-ninety-eight individuals were included in the study: 70 AD patients, 61 MCI individuals and 67 sex- and age-matched healthy controls (HC). All subjects were recruited by the Rehabilitative Neurology Unit of the IRCCS Fondazione Don Carlo Gnocchi, in Milan, Italy; the AD patients were enrolled in neurocognitive rehabilitation programs. Patients were diagnosed as having AD according to the National Institute of Neurological and Communicative Disorders and Stroke and the Alzheimer’s Disease and Related Disorders Association (NINCDS-ADRDA) criteria [19] or as having MCI according to Albert et al. criteria [3]. HC were selected from volunteers of IRCCS Fondazione Don Carlo Gnocchi, considering as exclusion criteria the presence of impairment in functional activities of daily living and/or psychiatric illnesses as assessed with a clinical interview.

The study conformed to the ethical principles of the Declaration of Helsinki; all subjects or their legal guardians gave informed and written consent according to a protocol approved by the local ethics committee of the IRCCS Fondazione Don Carlo Gnocchi. (#12_21/6/2018) For each subject, whole blood and serum samples were collected.

### 2.2. HSV-1 IgG Analyses

HSV-1 serum IgG titers were measured using commercial enzyme-linked immunosorbent assay (ELISA) (IBL International, Hamburg, Germany), according to standard protocol. The optical densities (OD) of wells were determined at 450/620 nm. HSV-1 Ab titers were expressed as antibody index (AI), calculated by dividing OD measurement generated from the assay by OD cut-off calibrator. Subjects with AI > 11 were seropositive, whereas subjects with AI <9 were seronegative. If the results were in grey zone (AI between 9 and 11), the tests were repeated in triplicate and, if the results remained in grey zone, the subjects were excluded from the study.

Quantitation of the HSV-1 IgG_3_ subclass was carried out by an opportunely-modified ELISA assay (HSV-1 IgG ELISA, Sigma-Aldrich, St. Louis, MO, US), and the results were expressed as the OD, as previously reported [16].

### 2.3. Apolipoprotein E (ApoE) Genotyping

Genomic DNA was isolated from whole blood from each subject by phenol–chloroform extraction. Customer-design Taqman probes for the 112 and 158 codons were used to determine the genotype of *ApoE* gene [20].

### 2.4. Morphometrical Analyses—MRI

To investigate gray matter morphometry, in close temporal proximity with blood sampling, a randomly-selected subgroup of AD (*n* = 40) and MCI (*n* = 35) individuals underwent a high-resolution 3D-T1 image acquired on a 1.5 Tesla scanner (Siemens Magnetom Avanto, Erlangen, Germany). Parameters of the high-resolution 3D-T1 (MPRAGE) image were TR/TE = 1900/3.37 ms, FoV = 192 mm × 256 mm, in-plane resolution 1 mm × 1 mm, slice thickness = 1 mm, and number of contiguous axial slices = 176. 3D-T1 images were analyzed using Freesurfer’s recon-all pipeline (v 5.3, https://surfer.nmr.mgh.harvard.edu/ [21]. Quality checks were performed according to the manual quality control procedure described in [22] and corrections were manually performed to improve automatic segmentation. In order to obtain total hippocampal volumes, the hippocampal subfield segmentation tool of Freesurfer (v.6.0) [23] was used and estimated total intracranial volume (eTIV) was also computed using Freesurfer automatic subcortical segmentation [24].

**Figure 1:**
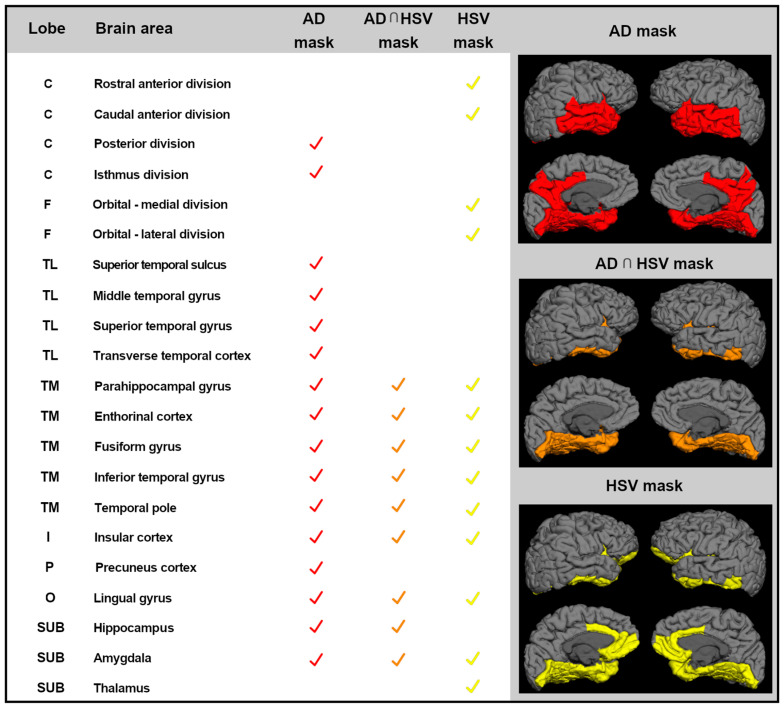
Ad-hoc masks of brain areas considered in the statistical analyses. List of all the brain areas included in each mask: in red color, brain regions that are target areas in the mild stages of AD pathology (AD mask); in yellow color, cortical areas that are the target for HSV-1 encephalitis (HSV-mask); and in orange color, the areas commonly shared by AD mask and HSV-mask (AD∩HSV-1-mask). AD: Alzheimer disease; C: cingulum; F: frontal; TL: temporal lobe, lateral aspect; TM: temporal lobe, medial aspect; I: insula; P: parietal; O: occipital; SUB: subcortical volumes.

To obtain gray matter atrophy measures, cortical parcellations were obtained for each subject according to Desikan atlas [25] and were used to obtain three ad hoc brain masks, created according to literature (see Figure 1). These masks were: (1) brain mask of AD target areas in the mild stages of the disease (AD-mask), including bilaterally-posterior cingulate cortex and temporal lobe areas (superior, middle, and inferior gyrus, medial temporal lobe areas, and total hippocampal and amygdala volumes) [26,27]; (2) brain mask of HSV-1 target areas (HSV-mask), including bilaterally-anterior cingulate and orbitofrontal cortices, medial temporal lobe areas, insula and total hippocampal, and thalamus and amygdala volumes according to [28,29,30,31]; and (3) a mask including the brain regions overlapping between AD-mask and HSV-mask brain masks (AD∩HSV-1-mask), including bilaterally-medial temporal lobe cortices and total hippocampal and amygdala volumes.

Morphometrical data (volumes/thickness) were extracted for each subject and included in subsequent statistical analyses: (a) to test downstream neuronal degeneration in our sample and (b) to test the relationship between morphometrical features and IgG_3_ Ab titers (see statistical analysis section).

### 2.5. Statistical Analysis

#### 2.5.1. Demographical and HSV-1-IgG Analyses

The parametric data are expressed as mean ± standard deviation, whereas the non-parametric data are expressed as median and interquartile range (IQR). Differences in experimental data among groups were tested using the Kruskal–Wallis test and, when appropriate, the Mann–Whitney U test, and the correlations, using Spearman’s correlation coefficient. *p*-values corresponding to ≤0.05 are described as statistically significant in the text. The statistical analyses were accomplished using commercial software (MedCalc Statistical Software version 14.10.2, Ostend, Belgium).

#### 2.5.2. Morphometrical Analyses—MRI

(a) To test downstream neuronal degeneration in our MRI subsample, an ANCOVA analysis was performed in order to compare hippocampal volume data of AD and MCI subjects with a template of age- and gender-matched healthy subjects from a dataset from our laboratory (HC-MRI) whose brain images had been acquired and analyzed with the same pipeline described above. Estimated TIV was inserted as covariate of no interest to account for differences in brain size. Statistical analyses have been considered as statistically significant when surviving *p* < 0.05 statistical threshold.

(b) To test the presence of relationship between IgG_3_ titers and morphometrical indices, statistical analyses were performed separately for AD and MCI subsamples considering hippocampal volumes and cortical thickness in the selected areas (AD mask, HSV-mask, AD∩HSV-1-mask) and including only those subjects with serum presence of IgG_3_ *(n* = 30 MCI and *n* = 27 AD). Partial correlations between IgG_3_ Ab titers and brain thicknesses were then computed using MedCalc software (v. 19.1). Age, gender, education, and mini mental state evaluation (MMSE) raw score were included as covariates of no interest. Partial correlations were also computed between IgG_3_ Ab titers and subcortical volumes (hippocampus, thalamus, and amygdala) also inserting eTIV as an additional covariate of no interest. Statistical analyses were considered as statistically significant when surviving *p* < 0.05 statistical threshold.

## 3. Results

### 3.1. Demographical and Clinical Characteristics of the Subjects

Gender, educational level, and age were comparable in the three groups examined; global cognitive levels (MMSE) were, as per definition, significantly reduced in AD and MCI subjects compared to HC (*p* < 0.0001) and in AD compared to MCI subjects (*p* < 0.0001). The presence of APOE ε-4 is recognized as strong risk factor for AD development; as expected, a significantly higher frequency of the APOE ε-4 variant was detected in AD and MCI subjects compared to HC (*p* < 0.05). None of the individuals enrolled in the study had suffered from HSV-1 encephalitis in the past; no evidence of a particularly severe herpes labialis was recorded in the anamnesis. Finally, no familiarity for AD or other neurodegenerative diseases was reported in any of the analysed subjects. Demographic and clinical characteristic of the individuals enrolled in the study and of those who underwent MRI analysis are summarized in Table 1.

### 3.2. HSV-1-Specific Antibody Titers

The frequency of HSV-1 IgG detection was similar in the study population (AD: 90 %; MCI: 95 %; HC: 88 %). As previously reported [15,16], HSV-1-specific IgG titers were significantly higher in AD (9.10; 7.22–10.11 AI; *p* = 0.003) compared to MCI patients (8.40; 6.25–9.77 AI; *p* ≤ 0.05) and HC (7.71; 6.16–8.59 AI) (Table 1). Moreover, HSV-1-specific IgG_3_ were more frequently detected in MCI patients (88%) compared to HC (66%, *p* = 0.008) and to AD patients (77%), whereas the serum concentration of these antibodies was similar in the three groups (AD: 0.66; 0.48-0.80; MCI: 0.66; 0.47–0.86; HC: 0.66; 0.53–0.88) (Table 1).

### 3.3. MRI Analyses

Among the cohort of AD and MCI patients for whom good quality MRI images were available, we included in the statistical analyses those subjects with detectable IgG_3_ in serum (*n* = 30 MCI and *n* = 27 AD). Demographical data and hippocampal total volumes are reported in Table 2 and compared to an internal dataset of age and gender-matched HC.

#### 3.3.1. Assessment of Downstream Neuronal Degeneration

The between-group ANCOVA comparison showed a group effect for bilateral hippocampi, with a progressive reduction with the more severe form of pathology (AD volume < MCI volume < AD volume) (Table 2).

Post-hoc comparison showed for bilateral hippocampal volume a significant (Bonferroni corrected) reduction in AD patients compared to HC-MRI (right hippocampus *p* < 0.0001, left *p* < 0.0001) and in AD compared to MCI patients (right hippocampus *p* < 0.008, left *p* < 0.003). The comparison between MCI and HCI-MRI did not survive statistical post-hoc threshold (right hippocampus *p* = 0.136, left *p* = 0.064).

#### 3.3.2. Correlations between Cortical Thickness and HSV-1-Specific Igg_3_

Partial correlation analyses were performed between morphometrical variables and Ab IgG_3_ titers separately for AD and MCI groups.

In the AD group, partial correlation analyses demonstrated the presence of statistically-significant inverse correlation between bilateral temporal cortices and IgG_3_ titers. In particular, such inverse correlation was observed in left middle temporal gyrus, parahippocampal gyrus, fusiform gyrus, inferior temporal gyrus, and temporal pole. Inverse correlations were also found in right hemisphere in superior temporal and transverse temporal gyri and in right temporal pole. No significant correlations were found in the subcortical areas considered as region of interest; these results are summarized in Table 3.

In the MCI group, finally, partial correlation highlighted the presence of inverse correlation between IgG_3_ titers and left isthmus of cingulum and right superior temporal and transverse temporal gyri (Table 3).

## 4. Discussion

The involvement of HSV-1 in AD was first hypothesized in 1982, when the memory loss and cognitive impairment observed in herpes simplex encephalitis (HSE) patients were noticed to be associated with alterations of the same brain areas as those involved in AD (limbic system and frontal and temporal cortices) [32]. From that first claim, several studies investigated this hypothesis, supporting the evidence of a dissemination of HSV-1 in the brain, with a specific neurotropism strongly associated to AD (see [6] for a review). The detection of viral genome in AD brains has been reported previously [7,33,34,35], although not always confirmed [36,37,38,39]. The demonstration of viral replication or transcriptional activity in AD brains has yet to be demonstrated. Notably though, recent data obtained in the mouse model showing that after recurrent reactivation HSV-1 can reach the brain and can produce accumulation of neurodegeneration leading to impairment [10] offers further support to the hypothesis that HSV-1 plays a pathogenetic role in AD.

The present study analyzed the relation between serum HSV-1-specific IgG_3_ antibodies and the damage observed in brain of AD and MCI subjects. This approach stems from the previously-observed differences in HSV-1-specific humoral response in AD, MCI and age-and-sex-matched HC individuals [14,15], and in particular from the findings showing that HSV-1 specific IgG_3_ is more frequently observed in MCI subjects compared to AD and healthy controls [16]. IgG_3_ is endowed with the strongest neutralization ability in response to pathogens [40] and plays a very important role against HSV-1 infection, as this subclass is the only one that is not recognized by the vFcR, a viral receptor expressed by HSV-1 that allows virus evasion from the host immune response [17].

Herein results confirm our previous findings: although HSV-1 seroprevalence is similar among the analyzed groups, serum concentration of HSV-1-specific IgG antibodies is increased in AD and MCI subjects compared to controls. Despite serum concentration of HSV-1- specific IgG_3_ being similar among the three groups, this subclass of antibody was more frequently detected in MCI individuals compared to AD patients and HC. We have previously shown that HSV-1-specific neutralizing activity of serum from AD patients is reduced compared to that of MCI individuals or HC, independently of the concentration of IgG_3_. To further analyze possible correlations between HSV-1-specific immune responses and AD –associated neurodegeneration we next correlated IgG_3_ titers and cortical thickness of brain areas that are characteristically altered in AD pathogenesis.

Considering as regions of interest those brain areas where neurodegeneration typically occurs in AD (bilaterally posterior cingulated cortex and temporal lobe areas), results of these analyses showed the presence of a significant negative correlation between HSV-1-specific IgG_3_ serum concentration, brain volumes, and cortex thinning in bilateral temporal cortices in AD patients. Interestingly, this highly-significant correlation involved brain areas typically altered in AD and also extensively targeted in HSV-1 encephalitis (left temporal pole and parahippocampal gyrus). Results obtained in MCI individuals, showed the presence of negative correlations between HSV-1-specific IgG_3_ serum concentration and the volumes of the cingulum and the right superior temporal gyrus and transverse temporal cortex, regions that are involved in dementia [26,27] but not in HSV-1 encephalitis [28,29,30,31].

A possible interpretation of the different results observed in AD and MCI patients is that in MCI the immune system is still relatively preserved; IgG_3_—the only Ab subclass that cannot be evaded by HSV-1—are more frequently expressed in this prodromal step of AD. This would contain recurrent HSV-1 replications and reduce brain damage, slowing down the progression of the disease, at least for a while. In AD patients, on the contrary, as time progresses, the immune system is impaired [41,42] and HSV-1-specific immune responses fail to control viral replication in the CNS, leading to a spreading of the damaged brain area. This hypothesis is supported by our previous results, showing that AD patients’ serum is characterized by a defective HSV-1-specific neutralizing activity even in the presence of high IgG_3_ levels [16].

Results herein show that in AD, but not in MCI, increased IgG_3_ titers are associated with increased brain damages. Taken together these finding suggest that the ability of IgG_3_ to neutralize HSV-1 is reduced with progression of disease from MCI to AD.

## 5. Conclusions

These data reinforce the hypothesis that HSV-1 plays an important role as a co-factor in AD and confirm that the progression of disease can be associated to a complex and multifactorial scenario that includes, beside genetic pattern, host immunity and environmental agents.

## Figures and Tables

**Table 1:** Demographic, clinical and virological characteristics of subjects enrolled in the study.

Parameters	AD	MCI	HC	*p*-Value
**N.**	70	61	67	
**Gender (M:F)**	28:42	30:31	28:39	n.s.
**Age, years**	75.8 ± 6.4	74.0 ± 5.6	72.3 ± 7.8	n.s.
**MMSE**	20.7 ± 3.2	25.1 ± 2.1	29.3 ± 0.5	<0.0001
**Level of education, years**	8.7 ± 3.6	9.7 ± 4.3	8.6 ± 3.9	n.s.
**APOE ε-4 carriers (%)**	44	36	18	<0.05
**HSV-1-specific IgG, % and AI**	90% 9.1; 7.2–10.1	95% 8.4; 6.2–9.8	88% 7.7; 6.2–8.6	n.s. <0.05
**HSV-1-specific IgG_3_, % and OD**	77% 0.6; 0.5–0.8	88% 0.6; 0.5–0.9	66% 0.6; 0.5–0.9	<0.05 n.s

Data are reported as mean ± standard deviation or as median and interquartile range. AD: Alzheimer’s disease; MCI: mild cognitive impairment; HC: healthy controls; M: male; F: female; MMSE: mini mental state evaluation; APOE: apolipoprotein E; AI: antibody index; OD: optical density.

**Table 2:** Demographical data and hippocampal volume in the selected subgroup of AD and MCI subjects with serum presence of IgG_3_, in comparison with a template of healthy subjects (HC-MRI).

Parameters	AD	MCI	HC-MRI	*p*-Value
**N.**	27	30	33	
**Gender (M:F)**	10:17	15:15	9:24	n.s.
**Age, years**	75.8 ± 5.6	72.9 ± 6.2	72.7 ± 5.0	n.s.
**MMSE**	22.6 ± 0.3	26.7 ± 0.3	29.2 ± 0.3	< 0.001
**Right hippocampal volume**	2592.8 ± 77.3	2922.5 ± 73.3	3129.1 ± 70.1	< 0.001
**Left hippocampal volume**	2435.3 ± 83.9	2828.3 ± 79.6	3087.5 ± 76.1	< 0.001

Data are reported as mean ± standard deviation. AD: Alzheimer’s disease; MCI: mild cognitive impairment; HC: healthy controls; M: male; F: female; MMSE: mini mental state evaluation.

**Table 3:** Relationship between morphometrical variables and IgG_3_ titers.

Lobe	Brain Area		MCI		AD		AD Mask	AD ∩ HSV	HSV Mask
			Left	Right	Left	Right			
C	Isthmus division	r	−0.467	0.07561	−0.2187	−0.3781	x		
		*p*-value	0.0162 *	0.7136	0.3162	0.0752			
TL	Middle temporal gyrus	r	−0.2268	−0.1363	−0.5654	−0.1744	x		
		*p*-value	0.2653	0.5067	0.0049 *	0.4262			
TL	Superior temporal gyrus	r	−0.2703	−0.496	−0.3303	−0.4252	x		
		*p*-value	0.1816	0.01 *	0.1237	0.0431 *			
TL	Transverse temporal cortex	r	−0.2483	−0.4874	−0.1729	−0.4337	x		
		*p*-value	0.2213	0.0115 *	0.4302	0.0387 *			
TM	Parahippocampal gyrus	r	0.0799	0.01633	−0.5272	−0.3543	x	x	X
		*p*-value	0.6981	0.9369	0.0097 *	0.0972			
TM	Fusiform gyrus	r	−0.3602	−0.3018	−0.4156	−0.1747	x	x	X
		*p*-value	0.0706	0.1341	0.0486 *	0.4254			
TM	Inferior temporal gyrus	r	−0.2018	−0.3054	−0.4832	−0.2864	x	x	X
		*p*-value	0.3228	0.1293	0.0195 *	0.1853			
TM	Temporal pole	r	−0.0795	−0.1542	−0.5661	−0.4616	x	x	X
		*p*-value	0.6996	0.452	0.0049 *	0.0266 *			

Statistically significant results of partial correlation analyses are showed (in bold and with *) in both MCI and AD patients. AD: Alzheimer’s disease; MCI: mild cognitive impairment; C: cingulum; TL: temporal lobe, lateral aspect; TM: temporal lobe, medial aspect.
